# Syringotropic mycosis fungoides with clinical and histologic features of necrobiosis lipoidica diabeticorum

**DOI:** 10.1016/j.jdcr.2024.02.029

**Published:** 2024-03-08

**Authors:** Mónica Torres-Piwinski, Kacie R. Carlson, Gauri Panse, Michael Girardi

**Affiliations:** a Universidad Central del Caribe School of Medicine, Bayamón, Puerto Rico; b Department of Dermatology, Yale School of Medicine, New Haven, Connecticut

**Keywords:** cutaneous T-cell lymphoma, folliculotropic, interstitial, mycosis fungoides, necrobiosis lipoidica diabeticorum

## Introduction

Mycosis fungoides (MF) is an extranodal non-Hodgkin lymphoma and the most common form of cutaneous T-cell lymphoma. The malignant T cells in MF show a propensity to home to and accumulate in the skin, and in later stages, may progress to the lymph nodes and peripheral blood. MF typically follows an indolent clinical course where malignant epidermotropic T cells manifest as cutaneous patches that over years may evolve into plaques and tumors.^1^ In the early stages, it may be challenging to distinguish the clinical and histologic manifestations of MF from benign inflammatory conditions. Relatively rarer dermal forms of MF, as in interstitial or granulomatous variants, may in particular be challenging to differentiate from granuloma annulare, morphea, and necrobiosis lipoidica diabeticorum (NLD).^2^^,^^3^ Careful examination of histologic biopsy specimens for the presence of atypical lymphocytes and epidermotropism, as well as molecular analysis for T-cell clonality, eg, via polymerase chain reaction of T-cell receptor gene rearrangements, are warranted in suspected inflammatory conditions as these features may be indicative of variants of MF. Further complicating accurate diagnosis of dermal forms of MF, malignant T cells in MF may exhibit tropism for adnexal structures, as seen in syringotropic mycosis fungoides (STMF) and folliculotropic mycosis fungoides (FMF). As each affects various components of pilosebaceous and eccrine units, STMF and FMF are clinically similar with the caveat that STMF may particularly involve eccrine-rich areas and shows histologic features of hyperplastic eccrine glands with atypical lymphocytic infiltrates within the dermis.^4^ Distinction is nonetheless important, as STMF typically follows a less aggressive clinical course than FMF.^4^ Herein, we present a rare report of a 64-year-old man with STMF manifesting a spectrum of clinical and histopathologic findings commonly found in NLD.

## Case Report

**Fig 1:**
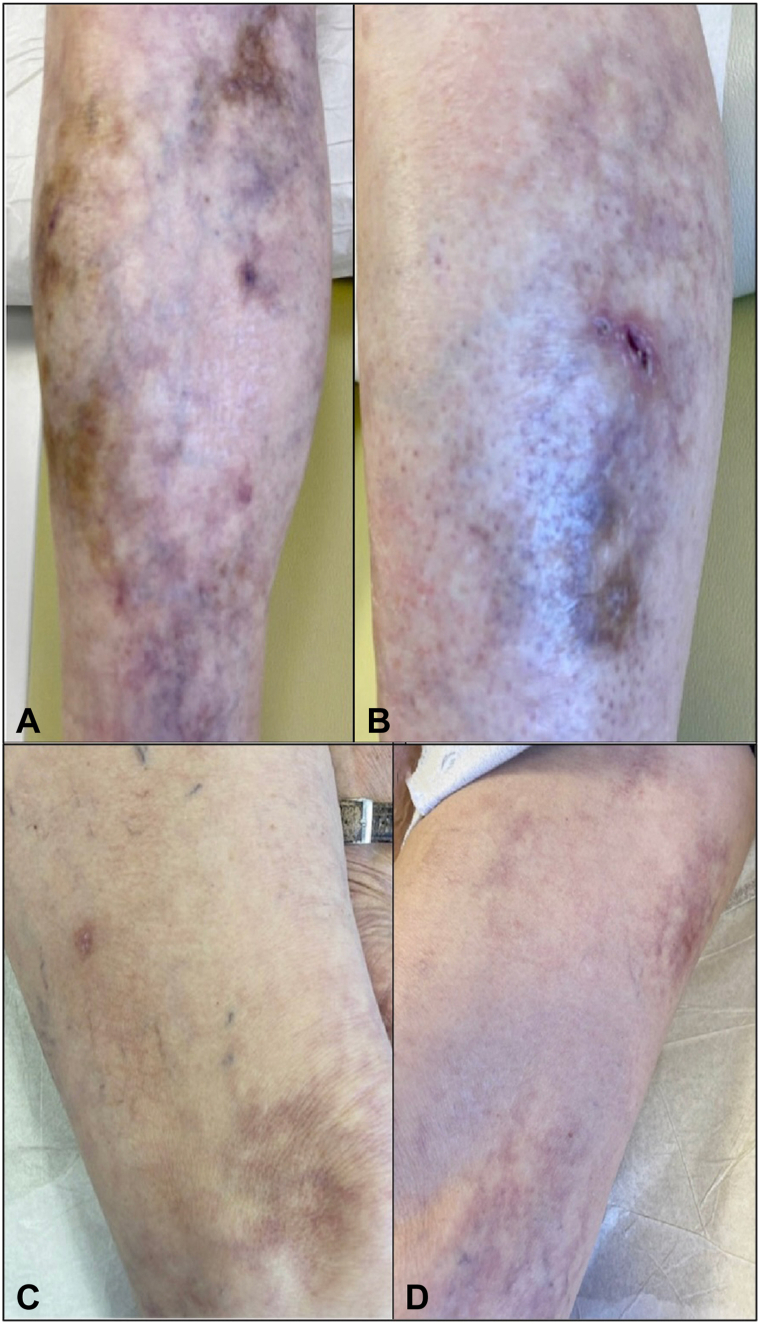
**A,** Right shin showed multiple brown-to-violaceous reticulated patches and thin plaques. **B,** A close-up of a similar shiny plaque on the left shin showed changes suggesting atrophy and follicular prominence. **C,** Yellow-brown thin plaques on the left thigh. **D,** Violaceous-to-brown irregular patches on the right thigh.

A 64-year-old White man presented with a 2-year history of a violaceous-to-brown patch on the right shin (Fig 1, *A*). Cutaneous biopsy was performed and histology showed a band-like lymphocytic and granulomatous inflammatory infiltrate with focal necrobiosis, compatible with NLD. There was also noted focal syringotropism that raised some concern for adnexotropic MF, although this was not considered diagnostic. Over approximately 2 years, multiple similar shiny patches on bilateral lower extremities emerged over the bilateral thighs and shins (Fig 1, *A*, *B*), and progressed to a mixture of pink to violaceous reticulated patches, brown shiny plaques, and faint pink oval patches with fine scale particularly concentrated on the medial thighs (Fig 1, *C*, *D*). Laboratory values on complete blood count and chemistry panel were within normal limits except for a fasting glucose level of 124 mg/dL, and flow cytometry was without phenotypically abnormal T cells. Livedo reticularis, superficial induration, and slight crusting noted in some areas prompted laboratory screening for antineutrophil cytoplasmic antibody (negative), cryoglobulins (negative), and phosphatidylserine/prothrombin (PS/PT) antibodies (positive at 69; reference range, <9).

**Fig 2:**
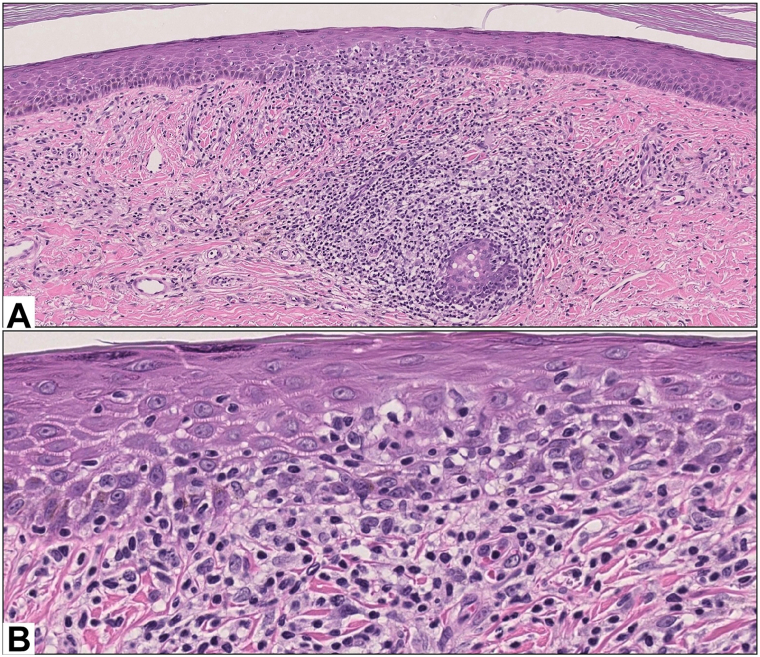
Low magnification **A,** and high magnification **B,** hematoxylin-eosin–stained sections from the right shin showed areas of epidermotropism and lymphocytic atypia.

**Fig 3:**
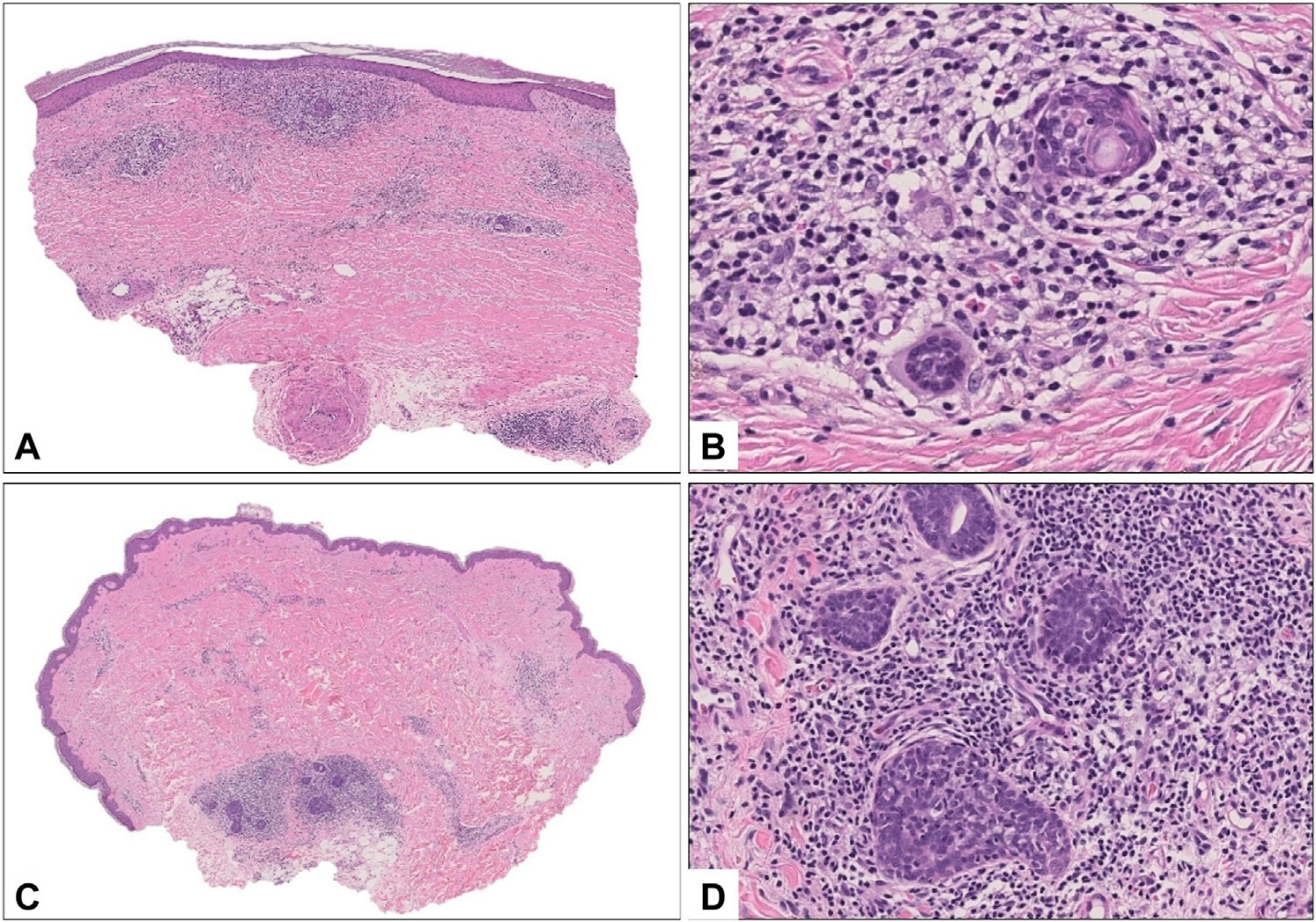
Hematoxylin-eosin–stained sections from right thigh showed a band-like lymphocytic and focally palisaded granulomatous infiltrate, mimicking necrobiosis lipoidica **A,** with foci of hyperplastic eccrine units containing lymphocytes **B**. **C, D,** Another biopsy showed lymphocytes infiltrating hyperplastic eccrine glands, characteristic of syringotropic or adnexotropic mycosis fungoides.

A second skin biopsy from the right shin NLD-like lesion was obtained to reveal foci of epidermotropism by lymphocytes exhibiting moderate cytological atypia (Fig 2). Additional skin biopsy samples were obtained from patches and plaques of the bilateral medial thighs and right side of the upper abdomen, and in all cases histopathology was notable for a dermal perifollicular and perieccrine infiltrate composed of slightly enlarged lymphocytes. The lymphocytes were accentuated around hyperplastic eccrine coils and adnexotropism was noted (Fig 3). The NLD-like lesion on the right shin showed T-cell clonal expansion identified by 2 polymerase chain reaction peaks within on primary set (V1-8) that was matched to the clonal peaks observed in 2 other specimens showing STMF histology: 1 from the thigh and 1 from the abdomen. The diagnosis was consistent with STMF with some areas showing clinical and histopathologic features of NLD (Fig 3).

## Discussion

Syringotropic mycosis fungoides (STMF) is a relatively rare variant of MF that affects ∼0.001% to 0.003% of the US population with an estimated incidence of 3.6 per million.^4^ STMF is characterized histopathologically by a dense dermal infiltrate of atypical lymphocytes surrounding and infiltrating hyperplastic eccrine glands.^4^ Clinically, STMF may present as either a single or multiple erythematous infiltrated papules, plaques, and/or patches that may display distinct studded comedo-like nodules. Typically, the anatomic distribution of STMF favors the extremities and in particular the palms or soles, whereas more classical MF typically arises on the sun-protected areas of the body. FMF may show similar follicular involvement to STMF, but without the eccrine predilection. Our patient’s histologic findings revealed hyperplastic eccrine coils infiltrated with lymphocytes, whereas 1 sample on the right medial thigh showed perifollicular lymphocytic infiltrates, with predominantly lymphocytic infiltrations supporting the diagnosis of STMF. T-cell clonality with matched clones across the variety of lesions confirmed the diagnosis.

STMF often requires aggressive treatment strategies including local radiotherapy or systemic therapies including retinoids, combination psoralen, and UV-A, and VELP (vincristine sulfate, etoposide, l-asparaginase, and prednisone acetate) chemotherapy.^5^ Our patient was initiated on oral bexarotene (150 mg daily) which has been previously reported for the treatment of both advanced MF and STMF.

The histopathology of NLD typically reveals interstitial palisaded granulomas with histiocytes, lymphocytes, plasma cells, and eosinophils,^6^ all present in the shin lesions of our patient. Nonetheless, within these lesions and a spectrum of others on the medial thighs and abdomen, histologic features of atypical lymphocytes, epidermotropism, and adnexotropism (Fig 3), as well as matched T-cell clonality shared across lesions, provides for a unifying diagnosis of STMF with features of NLD. Associated with diabetes mellitus, benign NLD is a chronic granulomatous disorder likely due to microangiopathic changes that ultimately lead to collagen degeneration.^6^ Although our patient did not have frank evidence of diabetes or vasculitis, he did show a slightly elevated fasting glucose level and positive antiphospholipid antibody, respectively, that may have contributed to the NLD-like changes and livedo reticularis.

Chronic cutaneous inflammatory states, eg, discoid lupus erythematosus,^7^ have largely been considered a tumor-promoting factor for keratinocyte-derived squamous cell carcinoma, but may also play a role in lymphomagenesis.^8^ The evidence for malignant transformation within a lesion of NLD lesion is limited to case reports and keratinocyte-derived cancer.^9^ Although our patient’s initial NLD-like skin lesions eventually were confirmed as STMF by histology and molecular analysis, it is unclear whether a bona fide benign NLD may have provided an inflammatory microenvironment supportive of lymphocyte transformation or more simply served as a locus minoris resistentiae for emerging STMF.

## Conflicts of interest

None disclosed.
